# Fuelling phytoremediation: gasoline degradation by green wall systems—a case study

**DOI:** 10.1007/s11356-023-30634-1

**Published:** 2023-11-02

**Authors:** Stephen Matheson, Robert Fleck, Thomas Lockwood, Raissa L Gill, Peter J Irga, Fraser R Torpy

**Affiliations:** 1 Plants and Environmental Quality Research Group (PEQR), School of Life Sciences, Faculty of Science, University of Technology Sydney, Ultimo, Australia; 2 Hyphenated Mass Spectrometry Laboratory (HyMaS), School of Mathematical and Physical Sciences, Faculty of Science, University of Technology Sydney, Ultimo, Australia; 3 Productive Coasts, Climate Change Cluster, Faculty of Science, University of Technology Sydney, Ultimo, Australia; 4 Plants and Environmental Quality Research Group (PEQR), School of Civil and Environmental Engineering, Faculty of Engineering and Information Technology, University of Technology Sydney, Ultimo, Australia

**Keywords:** Phytoremediation, VOCs, Green wall, Indoor air quality, Gasoline vapour

## Abstract

The capacity for indoor plants including green wall systems to remove specific volatile organic compounds (VOCs) is well documented in the literature; however under realistic settings, indoor occupants are exposed to a complex mixture of harmful compounds sourced from various emission sources. Gasoline vapour is one of the key sources of these emissions, with several studies demonstrating that indoor occupants in areas surrounding gasoline stations or with residentially attached garages are exposed to far higher concentrations of harmful VOCs. Here we assess the potential of a commercial small passive green wall system, commercially named the ‘LivePicture Go’ from Ambius P/L, Australia, to drawdown VOCs that comprise gasoline vapour, including total VOC (TVOC) removal and specific removal of individual speciated VOCs over time. An 8-h TVOC removal efficiency of 42.45% was achieved, along with the complete removal of eicosane, 1,2,3-trimethyl-benzene, and hexadecane. Further, the green wall also effectively reduced concentrations of a range of harmful benzene derivatives and other VOCs. These results demonstrate the potential of botanical systems to simultaneously remove a wide variety of VOCs, although future research is needed to improve upon and ensure efficiency of these systems over time and within practical applications.

**Supplementary Information:**

The online version contains supplementary material available at 10.1007/s11356-023-30634-1.

## Introduction

Urban VOCs are a group of carbon-based chemicals that consist largely of anthropogenic pollutants, such as vehicle emissions or gaseous by-products of synthetic materials and cleaning products (Irga et al. 2018; Jia et al. 2008). Commonly, indoor VOC concentrations exceed ambient outdoor air by 3–5 times, which is especially problematic given the low air exchange rates typical of contemporary buildings (Jafari et al. 2015). Indoor VOCs below the threshold for human perception are often associated with idiopathic discomfort, irritation and/or respiratory disease (Mitchell et al. 2007). At high concentrations, VOCs have been linked to reduced attention and performance, known as ‘sick building syndrome’, which may develop into a diverse range of pathologies with chronic exposure, including cancer, asthma and heart disease (Bernstein et al. 2008; Torpy et al. 2018a). On a daily basis, indoor occupants are exposed to one of many harmful aromatic hydrocarbons simultaneously, particularly those belonging to one of the four major pollutants derived from gasoline: benzene, toluene, ethylbenzene and xylene (BTEX) vapours (Al-Harbi et al. 2020).

Several studies have shown that indoor occupants of areas surrounding gasoline stations are exposed to higher levels of BTEX compounds, which in some cases exceed guidelines set by the World Health Organisation (WHO) (Abdo et al. 2016; Godoi et al. 2013; WHO 2010); the concentrations of airborne benzene associated with an excess lifetime risk of 1/10,000, 1/100,000 and 1/1,000,000 are 17, 1.7 and 0.17 μg/m^3^, respectively. An epidemiological study by Neghab et al. (2015) detailed the hepatic effects of BTEX inhalation by gasoline station employees, including subclinical or prepathological changes in liver and kidney function. The components of BTEX are known to induce acute non-lymphocytic leukaemia and myeloid leukaemia and have been associated with increased risk of chronic lymphocytic leukaemia and multiple myeloma in humans (Hamid et al. 2019; Warden et al. 2018). Edokpolo et al. (2014) conducted a literature review on BTEX exposure from 16 countries over 20 years and concluded that gasoline station workers exposed to between 1.9 and 2900 μg/m^3^ of benzene (~0.6 to 896 ppb at 21°C) were at the greatest risk of cancer. While BTEXs in the vicinity of gasoline stations are known to be elevated, exposure may not be limited to their immediate geographical location (Terrés et al. 2010). Demirel et al. (2014) recorded mean BTEX concentrations of 2.29, 26.55, 0.73 and 0.82 μg·m^−3^, respectively, inside urban schools which were up to 250 m away from the emission source. Furthermore, a study by Karakitsios et al. (2007) observed a 3–21% increased risk of cancer for people living within a 250 m vicinity of gasoline stations. Additionally, outside of BTEX, gasoline vapour emissions also include other benzene derivatives, alkanes and alicyclic hydrocarbons, with associated adverse health effects ranging from mild mucosal irritation to visible impacts on the central nervous system such as slurred speech and confusion (Micyus et al. 2005; Odewabi et al. 2014; Yue et al. 2017).

Of possibly greater but underrepresented concern is the presence of fuel vapour in residential buildings. Many studies have shown that VOC concentrations are considerably higher in residences with garages directly attached to the indoor living space (Batterman et al. 2007; Mann et al. 2001; Thomas et al. 1993; Tsai and Weisel 2000). Attached garages have also been identified as the single major source of benzene exposure to non-smoking individuals in such residences (Batterman et al. 2007). Emissions are significant for gasoline, diesel- and gas-powered engines and are especially concentrated during engine start-up (Wang et al. 2022), a process which occurs predominately in enclosed parking spaces. Furthermore, these exposures are not limited to residential buildings—many commercial spaces also have indoor parking facilities, not all of which feature appropriate emission-transfer mitigation systems (Batterman et al. 2007). These studies highlight the damaging nature of BTEXs in humans and demonstrate a lack of control mechanisms and protection for urban populations exposed to gasoline vapour.

Conventional air cleaning technologies such as the filters commonly used in heating, ventilation and air conditioning (HVAC) systems have been shown to be effective for particulate filtration (Chen et al. 2005). These systems draw in outdoor air via the building’s air handling unit (AHU), which is then passed through at least one HVAC filter comprising the AHU before being circulated indoors—effectively flushing the interior with ‘fresh’ outdoor air. While this process may reduce the build-up of interiorly sourced VOCs, HVAC systems are incapable of gaseous pollutant capture (Chen et al. 2005). Subsequently, the opportunity for outdoor origin pollutants such as fuel vapour to enter the indoor environment remains uncontrolled (Joshi 2008; Katsoyiannis and Bogdal 2012; Lawson et al. 2011). This can lead to problematic building occupant exposures at relatively low outdoor concentrations (Jafari et al. 2015). Fortunately, there is a rapidly growing body of literature documenting the capacity of plant-based systems to remove volatile organic compounds (VOCs) from indoor environments (Aydogan and Montoya 2011; Torpy et al. 2018b; Wolverton et al. 1984; Wood et al. 2006). For instance, Liu et al. (2022) observed 30.04% removal of total VOCs (TVOC) from cigarettes over a 12-h period, while Treesubsuntorn and Thiravetyan (Treesubsuntorn and Thiravetyan 2012) documented 45–77% removal of benzene over 72 h using *Dracaena sanderiana* Mast.

Plant systems remediate air contaminants by three different routes: removal through aerial parts of the plant and phyllospheric organisms (Wei et al.2017), removal by soil microorganisms (rhizosphere) and removal by the growing media (Aydogan and Montoya 2011). As our interest in the current work was to test functional effects that might be applied in situ rather than the mode of removal, our assessment methods recorded the combined effects of all of these removal methods. Green infrastructures such as green walls are emerging as an effective solution for the removal of air contaminants; green walls functionally act as a botanical biofiltration system which integrate plants along a vertical plane, which substantially increases the planting density and exposure of growth substrate to polluted air streams relative to conventional potted plant systems (Gunawardena and Steemers 2019). Rhizospheric bacteria and substrate adsorption are considered the primary sinks for pollutant removal within green wall systems (Dela-Cruz et al. 2023; Prodanovic et al. 2017; Prodanovic et al. 2018), whereas plant species followed by foliar uptake are secondary factors (Wood et al. 2006). Green walls have undergone *in situ* testing for indoor pollutant removal performance, demonstrating effectiveness for the reduction of both a broad range of VOCs and particulate matter (PM_2.5–10_) (Pettit et al. 2019a; Suárez-Cáceres et al. 2020). Active systems (i.e. using mechanical fans to provide airflow through the substrate) have demonstrated substantially higher air purification rates than passive systems (Irga et al. 2018), where active airflow exposes the biological components of the system to a greater volume of air, particularly the rhizospheric microbes in the growth substrate which remove VOCs by microbial degradation (Pettit et al. 2019a).

Most studies on VOC phytoremediation have assessed the removal of single VOC species, largely using analytical grade chemicals, and have documented how various individual indoor plant species (for an extensive list see Matheson et al. 2023) facilitate VOC removal (Aydogan and Montoya 2011; Dela Cruz et al. 2014; Kim et al. 2010; Pettit et al. 2019a). In reality, indoor occupants are exposed to a complex mixture of VOC species; this avenue of research remains understudied. Further, due to the requirement for mechanical systems for the introduction of airflow to active systems along with the attendant ducting and power supply, their costs are significantly higher than passive systems in all cases, making them a solution currently limited mostly to commercial applications. Therefore, passive green infrastructure may provide a favourable solution in a broad range of indoor environments. Passive green walls remove gaseous pollutants using the same biological mechanisms as active green wall systems, however at a slower rate as they lack active air flow provided by mechanical systems (Torpy et al. 2017); this absence of mechanical systems allows for cheaper installation and maintenance. Passive systems also feature simpler space and infrastructure requirements within indoor environments, are more widely available and provide an aesthetic that is appealing in many modern buildings, along with subsidiary services such as their well-described positive effects on mental health and wellbeing (Doxey et al. 2009; Han and Ruan 2020). While previous studies on passive systems have demonstrated proof of concept for the removal of common indoor pollutants (Aydogan and Montoya 2011; Dela Cruz et al. 2014; Kim et al. 2014; Teiri et al. 2018; Wolverton and McDonald 1982), while the phytoremediation of several of the individual hydrocarbons that constitute gasoline vapour has been explored previously, there are few studies that have examined the phytoremediation of azeotropic VOC mixtures such as gasoline vapour, which would commonly be seen within in situ environments (Dela-Cruz et al. 2023). As it is known that the simultaneous removal of multiple VOCs can result in interactions in individual VOC removal rates (Orwell et al. 2006), quantifying the removal of realistic VOC mixtures, in particular focussing on the toxic components, is needed to further our understanding of the air-cleaning capacity of plants in situ. Thus, the current study aimed to quantify the removal potential of a small passive green wall system for gasoline vapour, as well as recording the degradation for speciated petrochemical-derived VOCs with the use of gas chromatography–mass spectrometry (GC-MS; ISQTM 7610 Single Quadrupole GC-MS, Thermo Fisher Scientific™). This study provides insight into one of the major sources of indoor air contaminants worldwide and contributes to our understanding of the use of passive plant-based systems to improve indoor air quality.

## Methodology

### Commercial small live green walls

**Fig. 1:**
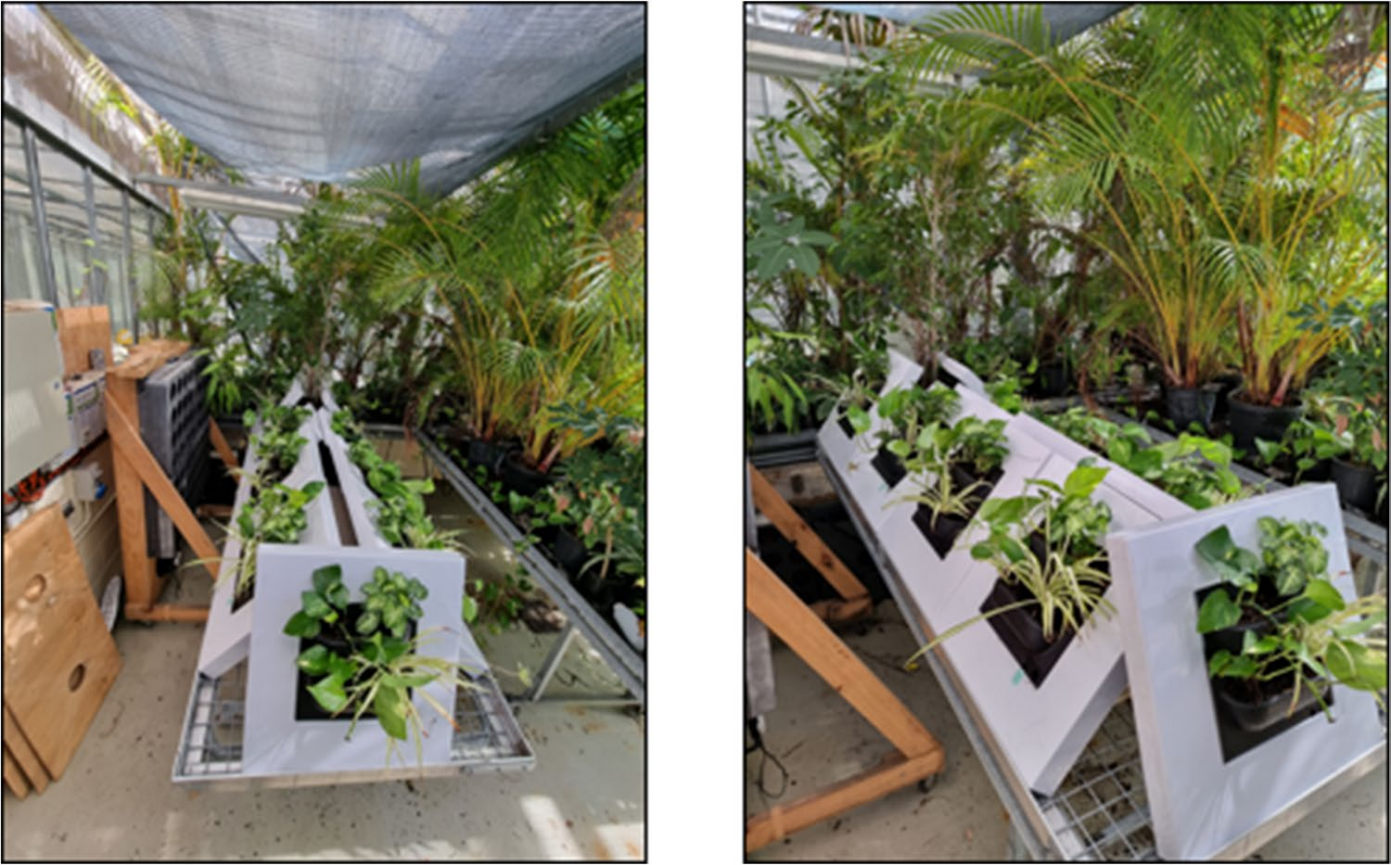
Experimental small-scale green wall biofilters (*n* = 9) maintained within a climate-controlled research glasshouse

Small-scale 725 mm × 725 mm commercial passive green walls were assessed (Ambius Small Live Green Wall, Ambius Pty Ltd Australia, Fig. 1). These systems contained 2 × 130 mm *Epipremnum aureum* (Linden ex André) G.S.Bunting, 1 × 130 mm *Syngonium podophyllum* Schott and 1 × 130 mm *Chlorophytum comosum* (Thunb.) Jacques. (Fig. 1) with a total leaf of 0.22 m^3^ ± 0.01 per green wall (plant species names are according to IPNI (http://www.ipni.org)) (International Plant Names Index (IPNI) 2023). These species were selected as they are all common indoor ornamental plants and have been studied in previous green wall research, showing potential to remove VOC concentrations (Sriprapat and Thiravetyan 2016). Each green wall unit contained 2.46 L of Hortico all-purpose blend potting mix (Dulux Group Australia Pty Ltd, Padstow, Australia) consisting of composted hardwood sawdust, composted bark fines and coarse river sand (2:2:1) (bulk density 0.6 gL^−1^; air-filled porosity 30%), with 1.25 g of slow release Osmocote total all-purpose fertiliser (Scotts Australia Pty Ltd, Baulkham Hills, Australia; N:P:K = 19.4:1.6:5). A watering mesh beneath the green wall substrate and a water reservoir (1 L capacity) were used to simplify maintenance: thus, all green wall units were maintained at a constant water level with a moisture content of 0.206 m^3^/m^3^. Green wall units containing solely potting-mix substrate with no plants were also tested and maintained as above to isolate the effect of the plants (‘substrate biofilter’ *n*=6). All green wall units were maintained in a research glasshouse prior to experimentation with a temperature of 23.7 ± 3.6 °C, relative humidity of 68.1 ± 16.0% and a maximum mid-day light level of 90 ± 10 μmol m^−2^ s^−1^ before and during experimentation.

### Experimental chamber set-up

All experimental trials were conducted in sealed Perspex chambers (0.6 × 0.6 × 0.6 m, 216 L) as previously described (Pettit et al. 2018). For gasoline vapour injections, the chambers were equipped with a 20 mm chlorobutyl septum and two DSII-8 probes with TVOC smart photoionization detectors (PID; Grey-Wolf, Ireland). TVOC sensors were calibrated by the manufacturer prior to use, with 1 ppb detection limit and <1 ppb degree of error. Environmental parameters including temperature, humidity and CO_2_ (DSII, Grey-Wolf, Ireland) were stable across all trials. Chambers were illuminated with light using two 41 W Parscan spotlights (ERCO Lüdenscheid, Germany) to simulate typical indoor lighting conditions (Dominici et al. 2021). This yielded 5–50 μmol m^−2^ s^−1^ of light on the leaf surfaces, where lower irradiances were detected on the shaded understory of the green wall plants. The CO_2_ concentration, temperature and humidity were consistent amongst all experimental trials, and thus any influence they might have had on VOC removal was eliminated from the experiment.

### Gasoline vapour trials

For TVOC testing, each green wall unit was placed in a sealed chamber and exposed to 1 mL of saturated (23°C) gasoline vapour over 8 h. Gasoline vapour was a composite mix of unleaded petrol sourced from three locations (1:1:1 ratio) within the Sydney metropolitan area. Two millilitre of the gasoline composite was placed into a sealed 5-mL vial and allowed to off-gas into the available headspace. The injection was introduced via a 5-mL gas tight syringe directly through the chlorobutyl septum and into the chamber, with an initial total concentration of 232 ± 35.70 ppb in the chamber space. VOC removal was quantified by chamber sensors (‘Experimental chamber set-up’ section).

**Fig. 2:**
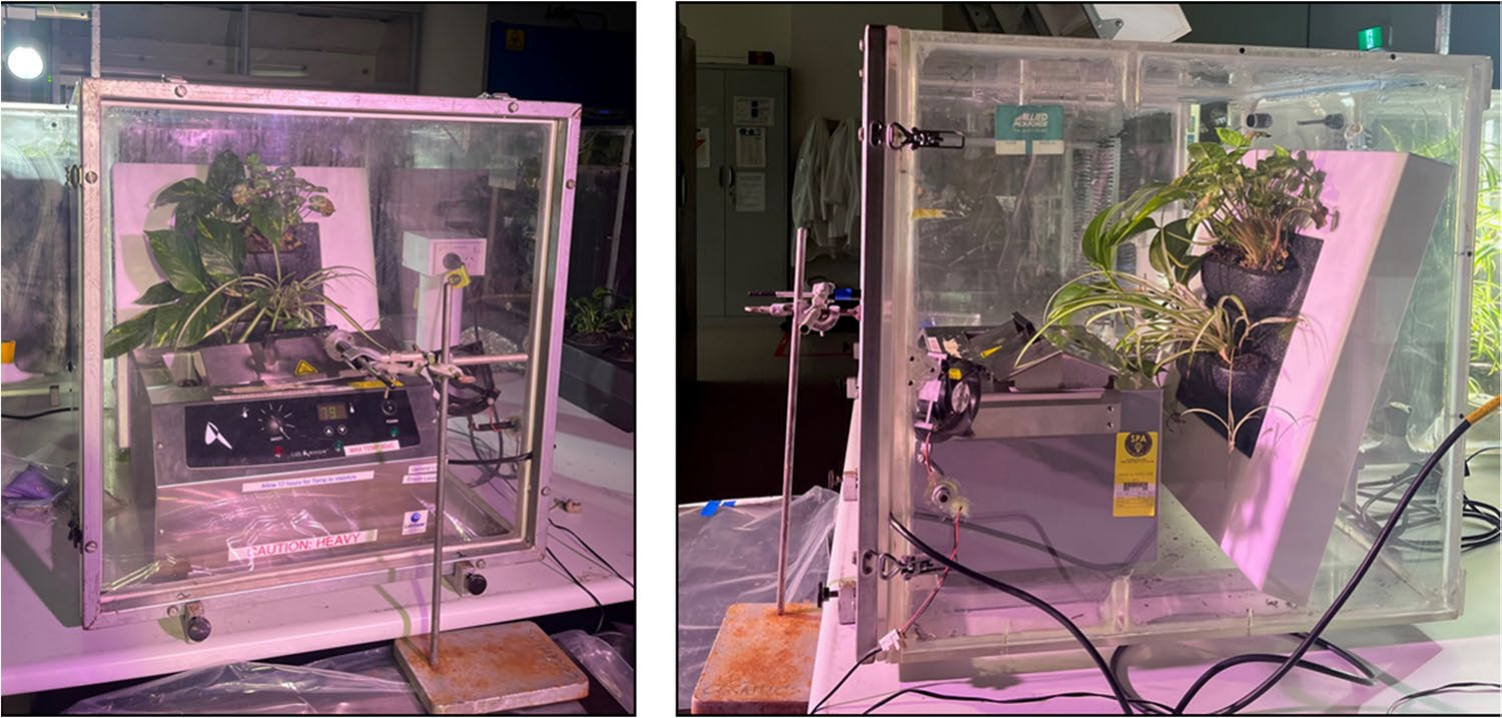
Experimental set-up for GC-MS speciation, including planted biofilter, heat bath for gasoline vapour generation and an SPME fibre for pVOC absorption

For speciated petrochemical VOC (pVOC) testing, 250 μL of the composite gasoline sample was placed in a heated bead bath (Sheldon manufacturing, USA) at 80°C and sealed in the chambers with the respective green wall unit. Gasoline samples evaporated completely within 30 min, at which point gas sample collection using solid-phase microextraction (SPME) fibres begun (Fig. 2). SPME fibres were composed of 30 μm polydimethylsiloxane (PDMS) which has been used previously to detect gasoline constituents in water (Kim et al. 2012). Directly after chamber sealing, a SPME fibre was inserted through the chlorobutyl septum and left for 1 h to allow pVOCs in the chamber space to adsorb. Each fibre was replaced every hour for an 8-h trial. After removal from the chambers, SPME fibres were analysed immediately by gas chromatography–mass spectrometry (GC-MS; ISQ™ 7610 Single Quadrupole GC-MS, Thermo Fisher, USA) to quantify specific pVOC removal.

For GC-MS analyses, fibres were desorbed in splitless mode for 1 min. pVOCs were separated in a HP-5MS capillary column (30 m × 0.25 mm ID, 0.25-μm film) using helium as the carrier gas at a flow rate of 1.0 mL/min. The injection and ion source temperature were 270°C with a MS transfer line temperature of 250°C. The column oven temperature was set at 40°C for 4 min, ramping to 150°C at a rate of 5°C per minute, and then at 40°C per minute to a final temperature of 220°C, which was held for 5 min. The total retention time was 32.75 min. Mass spectrometry was run with classical ionisation method with scans beginning after 1 min and a scan range of 40–200 atomic mass units (amu) and scan time of 0.5 s. Compounds were identified using the full scan mass spectra with a >85% similarity match to the National Institute of Standards and Technology (NIST) mass spectra library. The peak heights of each pVOC in the total ion chromatograph (TIC Height) were used to assess relative differences between treatments.

To address the plant specific VOC removal capacity, a substrate only control was assessed, where only the potting mix without plants was tested for pVOC removal. Additionally, to ensure the results were not confounded by chamber leakage, chemical degradation or adhesion to the chamber surfaces, an empty chamber control (‘no biofilter’ *n* = 6) was also employed. Combined, these two controls served to eliminate background VOC losses and allow us to quantify any non-plant specific variation in the data.

### Statistical analysis

**Fig. 3:**
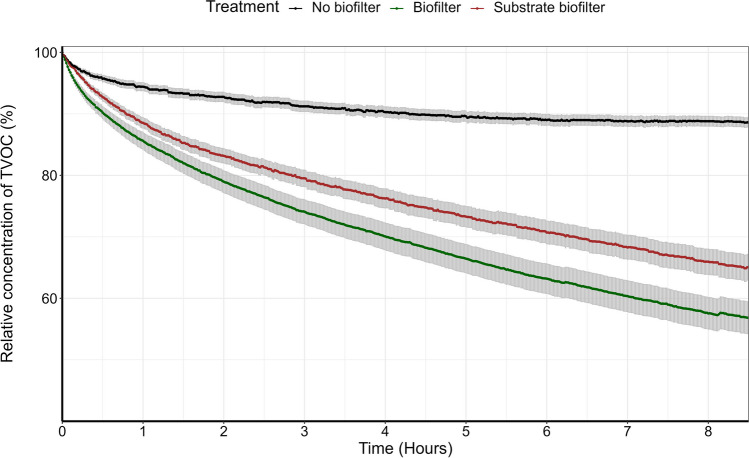
Relative TVOC drawdown for each treatment over 8 h. Error bands represent the SEM

**Fig. 4:**
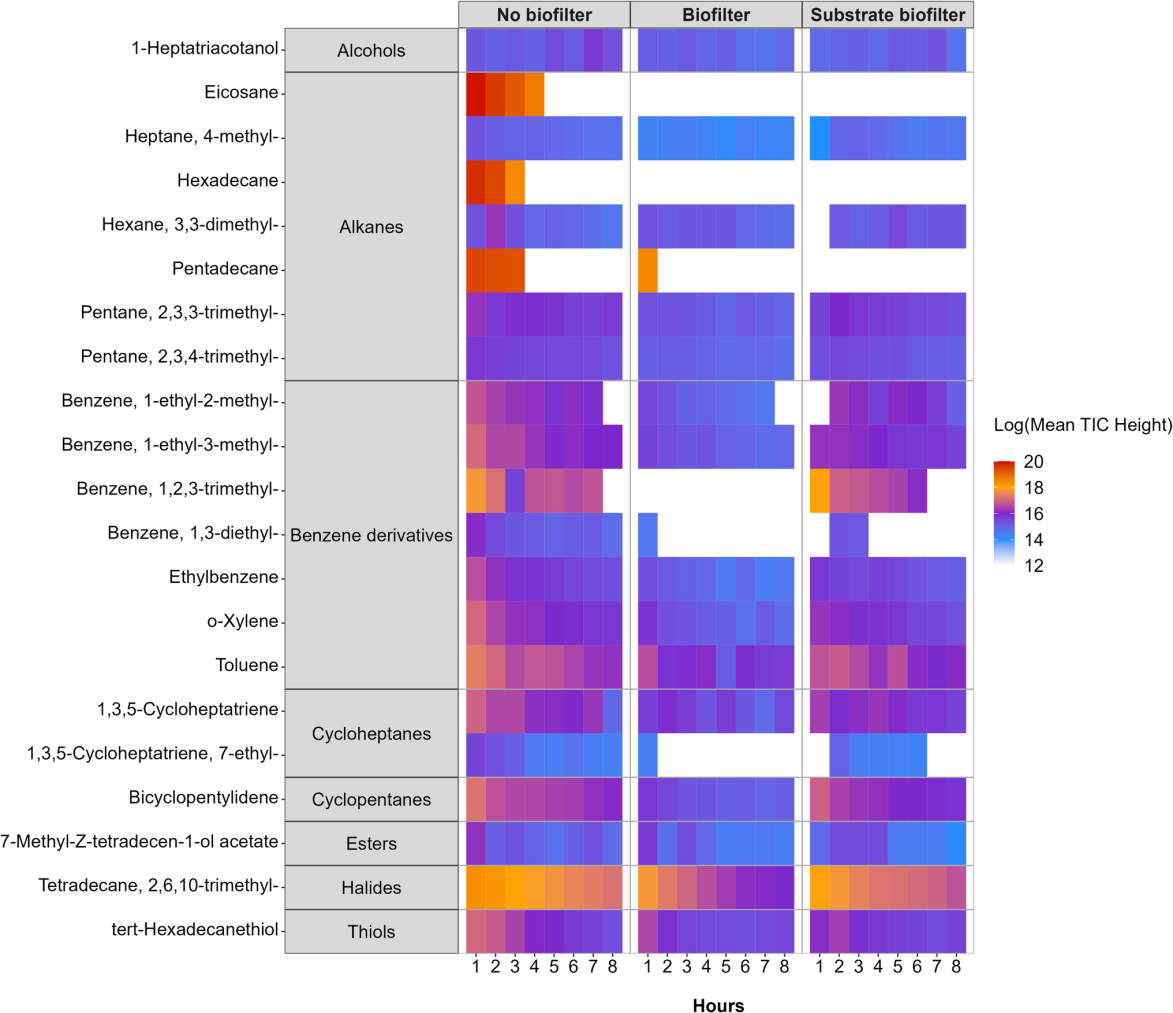
Speciated pVOCs identified in GC-MS analysis of each treatment. Grey boxes depict VOC functional groups. Each tile represents a timepoint, with its colour depicting the mean peak height (log scale), which is indicative of the remaining quantity of each VOC (for interpretation of the references to colour in this figure legend, the reader is referred to the Web version of this article)

Relative decay curves were generated by plotting TVOC injection concentrations over time and fitting each with a polynomial function. Decay is expressed as percentage removal from an initial average concentration of 232 ppb (Fig. 3). Exponential decay rates were taken as the equation of the line. Differences in the rates between treatments were compared by permutational analysis of variance (PERMANOVA) using Euclidean distance and 999 permutations. For pVOC speciation trials, an analysis of similarity percentages (SIMPER) was used to identify which pVOC species were driving differences in the cross-VOC removal patterns between the no biofilter and biofilter treatments. SIMPER was performed at two time points (1 h, 8 h) on a Bray-Curtis dissimilarity matrix of TIC-H comprising all pVOC species. Heatmap graphics were created to visualise speciated pVOC removal across treatments, with each tile representing the log mean TIC-H at each timepoint (Fig. 4).

All statistical analysis and graphics were performed in R version 4.0.4 and used the following packages: dplyr (Müller 2022), ggplot2 (Wickham, 2014), ggpubr (Kassambara 2022), pairwiseAdonis (Martinez Arbizu 2017), tidyr (Wickham, 2020), vegan (Jari Oksanen 2022) and xlsx (Dragulescu, 2020).

## Results

The injection protocol yielded reasonably consistent chamber TVOC concentrations (232 ± 35.7 ppb) over all experimental trials. The no biofilter treatment produced a decay rate of 1.56 ± 0.31 ppb.h^−1^, which represents the combined effect of VOC adhesion to or absorption into the chamber, leakage through the chamber seals and/or other uncharacterised chemical processes. The contribution of these effects on VOC degradation was relatively small relative to the effect sizes of the biofilter and substrate biofilter treatments (Fig. 3). The TVOC decay rates for the biofilter and substrate biofilter treatments were 11 ± 0.97 and 8.20 ± 1.42 ppb.h^−1^, respectively. These two treatments removed 42.47 ± 2.57 and 34.04 ± 2.05% of injected TVOCs, respectively, over 8 h. Both the biofilter and substrate biofilter treatments removed TVOCs faster than the empty chamber control (*p* = 0.00 and 0.01, respectively); however no difference was observed between the biofilter and substrate biofilter treatments (*p* = 0.16; Fig. 3). Prior to this trial, we employed a pilot test for 22 h (Fig. S1) to assess potential re-emission of VOCs. TVOC removal reached 64.49 ± 1.76% and 59.77 ± 1.76% over 22 h for the biofilter and substrate biofilter treatments, respectively, and no re-emission was detected.

After 1 h of exposure, GC-MS speciation detected 21 unique pVOCs above a certainty of 85% in the no biofilter treatment and only 18 in the biofilter treatments (Fig. 4). This indicates that three pVOCs: eicosane, pentadecane and 1,2,3-trimethyl-benzene, were removed by the green walls to below the detection limit of the GC-MS within the first hour. These three pVOCs were the major contributors to the dissimilarity between the VOC removal patterns of the planted and control treatments within the first hour (*p* = 0.006, 0.037 and 0.036; respectively) and exhibited removal approaching 100%. Comparatively, the substrate biofilter only removed two pollutants completely within the first hour of exposure: ethylbenzene and 1-ethyl-2-methyl-benzene.

Over an 8-h exposure, the biofilter treatment removed several additional pVOCs across a range of functional groups compared to the no biofilter treatment (Fig. 4). Notably, the biofilter removed 80.82 ± 0.01% of benzene-1-ethyl-2-methyl (*p* = 0.001), 79.68 ± 0.19% ethylbenzene (*p* = 0.003) and 82.29 ± 0.14% σ-xylene (*p* = 0.035). While we observed considerable reductions in toluene (72.23 ± 2.82%), this pVOC did not contribute significantly to the overall dissimilarity in SIMPER analysis. Speciated pVOC removal of the biofilter treatment differed significantly from the substrate biofilters (*p* = 0.004), exhibiting higher removal rates for the following pVOCs: tetradecane, 2,6,10-trimethyl- (91.64 ± 0.78 vs 80.79 ± 3.13%, *p* = 0.004), bicyclopentylidene (88.18 ± 0.97% vs 74.26 ± 2.90%, *p* = 0.007), tert-hexadecanethiol (74.7 ± 2.82% vs 74.26 ± 4.39%, *p* = 0.002), 1,3,5-cycloheptatriene (79.41 ± 4.18% vs 73.77 ± 3.96%, *p* = 0.018), pentane, 2,3,3-trimethyl- (70.38 ± 3.13% vs 56.33 ± 6.00%, *p* = 0.035) and 1-heptatriacotanol (30.43 ± 8.45% vs 49.58 ± 8.52%, *p* = 0.028).

## Discussion

Here we demonstrate the potential of botanical biofiltration to reduce concentrations of total and speciated gasoline-derived pVOCs from airstreams, the concentrations achieved in this study were representative of those measured road-side within proximity of gasoline stations (Edokpolo et al. 2014), and therefore the effect here may translate to real-world scenarios. However, laboratory experiments are limited by the environmental variables such as static drawdown conditions and laboratory settings, in particular light and the inherently variable VOC concentrations encountered in situ (Dela Cruz et al. 2014). Therefore, future studies should aim to identify areas that are prone to gasoline vapour contamination and assess the efficacy of active or passive green wall systems for the removal of pVOCs in situ. The passive green wall removed TVOCs faster than the no biofilter control (Fig. 3), where three pVOCs (eicosane, hexadecane, 1,2,3-trimethyl-benzene) were removed rapidly within the first hour, with reductions in an additional three species (1-ethyl-2-methyl-benzene, ethylbenzene and σ-xylene) over the full 8 h (Fig. 4). Interestingly, the substrate biofilter removed a similar quantity of TVOCs as the planted biofilter over an 8-h period (Fig. 3), highlighting the effectiveness of the substrate alone. This performance is likely to decline over time due to the absence of plant life to support the pVOC degrading microorganisms within the substrate (Aydogan and Montoya 2011; Hörmann et al. 2018; Torpy et al. 2013). Contrastingly, for planted biofilters, removal rates have been shown to increase over time, as repeated pVOC exposure upregulates natural pVOC degrading bacteria (De Kempeneer et al. 2004; Khaksar et al. 2016; Setsungnern et al. 2017; Torpy et al. 2013; Treesubsuntorn et al. 2013). While TVOC removal rates were similar, the presence of plants did influence speciated pVOC removal, where the planted biofilter was more effective in removing tetradecane, 2,6,10-trimethyl-, bicyclopentylidene, tert-hexadecanethiol, 1,3,5-cycloheptatriene, pentane, 2,3,3-trimethyl- (Fig. 4).

Unexpectedly, neither the planted nor substrate biofilter contributed significantly to overall dissimilarity to the chamber pVOCs (Fig. 4). Given that toluene degradation has been observed in previous green wall studies exposed to azeotropic VOC mixtures (Kim et al. 2016; Morgan et al. 2022; Mosaddegh et al. 2014; Sriprapat et al. 2014), this is likely due its overall removal being less than those pVOCs which significantly contributed. We also observed that some pVOCs within the substrate biofilter treatment appeared in the chromatogram at the second time point, namely 7-ethyl-1,3,5-cycloheptatriene, 1,3-diethyl-benzene, 1-ethyl-2-methyl-benzene, and 3,3-dimethyl-hexane (Fig. 4). This may be attributed to degradation of higher molecular mass hydrocarbons, leading to the release of new VOCs (Eriksson et al. 1998). These were consequently degraded themselves thereafter (e.g. 1,3-diethyl benzene, Fig. 4) or in some cases were more resistant to biological metabolism and remained in the chamber atmosphere until the final hour (e.g. 3,3-dimethyl hexane, Fig. 4). This effect was not observed in the biofilter treatment, with continuous removal of all identified pVOCs across the whole 8-h testing period. While removal performance is expected to increase with time due to the rhizospheric microbial community (Mikkonen et al. 2018), further experimentation is needed to establish the long-term performance of this system, especially with multiple VOC sources which has not previously been trialled over repeated exposures.

Previous work with homogenous pVOC exposures found *C. comosum* to be the most efficient plant species for toluene and ethylbenzene removal, demonstrating ~77 and ~70% removal, respectively, over a 72-h period (Sriprapat et al. 2014). While the green wall system tested here was predominantly the same species (Fig. 1), TVOC removal is lower in the present work (Fig. 4). Nonetheless, the green walls outperformed previously tested potted plants (Sriprapat et al. 2014), with 73.42 ± 8.24 and 80 ± 2.15% removal of ethylbenzene and toluene, respectively, after 8 h (Fig. 4). The capacity of the passive green wall, within this work, to outperform potted systems is likely due to its vertically planted design (Fig. 1), increasing plant density and substrate exposure per unit volume of chamber atmosphere and per unit of footprint area, facilitating more pollutant-to-leaf and substrate surface area contact (Darlington et al. 2001; Wolverton and McDonald 1982).

Compared with azeotropic VOC mixtures like tobacco smoke, the current study observed higher TVOC removal rates (~43% in 8 h; Fig. 3) than previous observations of potted plants (~30% in 12 h; Liu et al. (2022)) and active green wall systems (~43% single pass removal efficiency in; Morgan et al. (2022)). However, these differences are likely impacted by laboratory conditions and VOC source, especially given the greater diversity of VOCs encountered by both Liu et al. (2022) and Morgan et al. (2022). Most recently, a study conducted by Dela-Cruz et al. (2023) aimed to assess the long-term removal of specific VOC compounds derived from gasoline by passive potted plants. *Hedera helix* was exposed to gasoline vapours for 21 days, producing removal efficiencies for target pollutants ranging between 25 and 32%. While these removal efficiencies are substantially lower than the ones reported here and were achieved over a longer exposure period, the greater planting density of the current study (four plants per biofilter and chamber volume) has likely contributed to this effect. Similar to the Dela-Cruz et al. (2023) study, here we present the TVOC drawdown of gasoline vapour as well as the individual gasoline-derived VOCs which were the driving force of this removal.

In previous research (Pettit et al. 2019b), we have detected relationships between the molecular weight and dipole moment of various VOCs and their removal rates, although this work tested active botanical biofilters rather than the passive system used in the current work. The VOCs present in gasoline vapour consist of immensely diverse functional groups (Lewis 2018), as such their dipole moments could not be determined from literature (Nelson et al. 1967); however we tested the correlation between 8 h removal rate and molecular weight for all pVOCs, finding no significant relationship (*p*=0.08). While this would appear to contradict our previous findings, it is possible that significant patterns could be detected with greater sample sizes necessitated by a dedicated research project. Further examining the relationship between chemical properties and VOC removal rates is thus of ongoing interest.

This study represents one of the first investigations to quantify the ability of passive botanical systems to reduce realistic concentrations of real-world azeotropic VOC mixtures and to quantify the removal efficiency for all detectable VOCs present within petrochemical vapour. Previous studies have assessed the efficiency of potted plants to remove individual constituents which are present in gasoline vapour; however no previous work has tested a VOC mixture of the complexity used in the current work (Kim et al. 2016; Kim et al. 2014; Mosaddegh et al. 2014; Sriprapat and Thiravetyan 2013). Testing azeotropic mixtures of VOCs is likely to represent a more realistic test of phytoremediation capacity, where interactions between VOC species may occur (Orwell et al. 2006), as was observed in this study (Fig. 4). These results show the capability of the biofilter to remediate benzene derivatives and TEX compounds found within gasoline vapour, a group of known class 1 and 2a carcinogenic VOCs which pose some of the greatest health risks to persons exposed (Edokpolo et al. 2014; Neghab et al. 2015). Future studies should aim to identify areas that are prone to gasoline vapour contamination and assess the efficacy of both passive and active green wall systems for the removal of pVOCs *in situ*. Such tests would be of value as many of the pVOCs detected in this work also exist in *in situ* indoor environments not exposed to gasoline vapour (Adams et al. 2001; Durmusoglu et al. 2010). This would provide insight on the real-world performance of passive green walls for phytoremediation and how the introduction of other indoor pollutants may affect TVOC removal.

## Conclusion

This study demonstrates the potential of a passive green wall system to remediate indoor gasoline-derived VOCs over an 8-h working day timeframe, including both total and speciated compounds. This was achieved to varying degrees depending on pVOC species (complete or partial degradation). Our findings show that both planted and substrate only biofilters were capable of TVOC removal, with similar speciated pVOC profiles. However, the performance of the substrate is likely to decline with time due to the absence of plant life to support VOC-degrading microorganisms within the substrate. This research has significant implications given the highly toxic nature of the BTEX group found within gasoline vapour, which pose considerable risks to human health.

### Supplementary Information

Below is the link to the electronic supplementary material.

ESM 1 **Figure S1**. Relative TVOC drawdown for each treatment over 22 h. Error bands represent the SEM (PNG 653 kb)

High resolution image (TIF 2.25 mb)

## Data Availability

The data sets generated during and/or analysed during the current study are available from the corresponding author on reasonable request.

## Abbreviations

AHU: Air handling unit
BTEX: Benzene, toluene, ethylbenzene and xylene
GC-MS: Gas chromatography–mass spectrometry
HVAC: Heating, ventilation, air and conditioning
PDMS: Polydimethylsiloxane
PM: Particulate matter
pVOCs: Petrochemical volatile organic compounds
SPME: Solid-phase microextraction
TIC: Total on chromatogram
TVOC: Total volatile organic compounds
VOCs: Volatile organic compounds
WHO: World Health Organisation

## References

[CR1] Abdo P, Huynh BP, Avakian V, Nguyen T, Gammon J, Torpy FR, Irga PJ (2016) Measurement of air flow through a green-wall module. In Proceedings of the 20th Australasian Fluid Mechanics Conference, AFMC 2016

[CR2] Adams JM, Constable JV, Guenther AB, Zimmerman P (2001). An estimate of natural volatile organic compound emissions from vegetation since the last glacial maximum. Chemos -Global Change Sci.

[CR3] Al-Harbi M, Alhajri I, AlAwadhi A, Whalen JK (2020). Health symptoms associated with occupational exposure of gasoline station workers to BTEX compounds. Atmos Environ.

[CR4] Aydogan A, Montoya LD (2011). Formaldehyde removal by common indoor plant species and various growing media. Atmos Environ.

[CR5] Batterman S, Jia C, Hatzivasilis G (2007). Migration of volatile organic compounds from attached garages to residences: a major exposure source. Environ Res.

[CR6] Bernstein JA, Alexis N, Bacchus H, Bernstein IL, Fritz P, Horner E, Li N, Mason S, Nel A, Oullette J (2008). The health effects of nonindustrial indoor air pollution. J Allergy Clin Immunol.

[CR7] Chen W, Zhang JS, Zhang Z (2005). Performance of air cleaners for removing multiple volatile organic compounds in indoor air. ASHRAE Trans.

[CR8] Darlington AB, Dat JF, Dixon MA (2001). The biofiltration of indoor air: air flux and temperature influences the removal of toluene, ethylbenzene, and xylene. Environ Sci Technol.

[CR9] De Kempeneer L, Sercu B, Vanbrabant W, Van Langenhove H, Verstraete W (2004). Bioaugmentation of the phyllosphere for the removal of toluene from indoor air. Appl Microbiol Biotechnol.

[CR10] Dela-Cruz M, Svenningsen NB, Nybroe O, Müller R, Christensen JH (2023). Removal of a complex VOC mixture by potted plants—effects on soil microorganisms. Environ Sci Pollut Res.

[CR11] Dela Cruz M, Christensen JH, Thomsen JD, Müller R (2014). Can ornamental potted plants remove volatile organic compounds from indoor air?—a review. Environ Sci Pollut Res.

[CR12] Demirel G, Özden Ö, Döğeroğlu T, Gaga EO (2014). Personal exposure of primary school children to BTEX, NO2 and ozone in Eskişehir, Turkey: relationship with indoor/outdoor concentrations and risk assessment. Sci Total Environ.

[CR13] Dominici L, Fleck R, Gill RL, Pettit TJ, Irga PJ, Comino E, Torpy FR (2021). Analysis of lighting conditions of indoor living walls: effects on CO_2_ removal. J Build Eng.

[CR14] Doxey JS, Waliczek TM, Zajicek JM (2009). The impact of interior plants in university classrooms on student course performance and on student perceptions of the course and instructor. HortScience.

[CR15] Durmusoglu E, Taspinar F, Karademir A (2010). Health risk assessment of BTEX emissions in the landfill environment. J Hazard Mater.

[CR16] Edokpolo B, Yu QJ, Connell D (2014). Health risk assessment of ambient air concentrations of benzene, toluene and xylene (BTX) in service station environments. Int J Environ Res Public Health.

[CR17] Eriksson M, Swartling A, Dalhammar G, Fäldt J, Borg-Karlson A-K (1998). Biological degradation of diesel fuel in water and soil monitored with solid-phase micro-extraction and GC-MS. Appl Microbiol Biotechnol.

[CR18] Godoi RH, Godoi AF, Junior SJG, Paralovo SL, Borillo GC, Barbosa CGG, Arantes MG, Charello RC, Rosário Filho NA, Grassi MT (2013). Healthy environment—indoor air quality of Brazilian elementary schools nearby petrochemical industry. Sci Total Environ.

[CR19] Gunawardena K, Steemers K (2019). Living walls in indoor environments. Build Environ.

[CR20] Hamid HHA, Latif MT, Nadzir MSM, Uning R, Khan MF, Kannan N (2019). Ambient BTEX levels over urban, suburban and rural areas in Malaysia. Air Qual Atmos Health.

[CR21] Han K-T, Ruan L-W (2020). Effects of indoor plants on air quality: a systematic review. Environ Sci Pollut Res.

[CR22] Hörmann V, Brenske K-R, Ulrichs C (2018). Assessment of filtration efficiency and physiological responses of selected plant species to indoor air pollutants (toluene and 2-ethylhexanol) under chamber conditions. Environ Sci Pollut Res.

[CR23] International Plant Names Index (IPNI) 2023. Accessed October 16, 2023. https://www.ipni.org/

[CR24] Irga P, Pettit T, Torpy F (2018). The phytoremediation of indoor air pollution: a review on the technology development from the potted plant through to functional green wall biofilters. Rev Environ Sci Biotechnol.

[CR25] Jafari MJ, Khajevandi AA, Najarkola SAM, Yekaninejad MS, Pourhoseingholi MA, Omidi L, Kalantary S (2015). Association of sick building syndrome with indoor air parameters. Tanaffos.

[CR26] Jari Oksanen, G. L. S., F. Guillaume Blanchet, Roeland Kindt, Pierre Legendre, Peter R. Minchin, R.B. O'Hara, Peter Solymos, M. Henry H, Stevens, E. S., Helene Wagner, Matt Barbour, Michael Bedward, Ben Bolker, Daniel Borcard, Gustavo Carvalho,, Michael Chirico, M. D. C., Sebastien Durand, Heloisa Beatriz Antoniazi Evangelista, Rich Fitzjohn, Michael Friendly, Brendan, Furneaux, G. H., Mark O. Hill, Leo Lahti, Dan McGlinn, Marie-Helene Ouellette, Eduardo Ribeiro Cunha, Tyler Smith, Adrian, & Stier, C. J. F. T. B. A. J. W. (2022). vegan: community ecology package. In https://cran.r-project.org/package=vegan

[CR27] Jia C, Batterman S, Godwin C (2008). VOCs in industrial, urban and suburban neighborhoods—part 2: factors affecting indoor and outdoor concentrations. Atmos Environ.

[CR28] Joshi SM (2008). The sick building syndrome. Indian J Occupat Environ Med.

[CR29] Karakitsios SP, Delis VK, Kassomenos PA, Pilidis GA (2007). Contribution to ambient benzene concentrations in the vicinity of petrol stations: estimation of the associated health risk. Atmos Environ.

[CR30] Kassambara, A. (2022). ggpubr: ‘ggplot2’ based publication ready plots. In https://cran.r-project.org/package=ggpubr

[CR31] Katsoyiannis A, Bogdal C (2012). Interactions between indoor and outdoor air pollution-trends and scientific challenges. Environ Pollut.

[CR32] Khaksar G, Treesubsuntorn C, Thiravetyan P (2016). Effect of endophytic Bacillus cereus ERBP inoculation into non-native host: potentials and challenges for airborne formaldehyde removal. Plant Physiol Biochem.

[CR33] Kim K, Kim H, Khalekuzzaman M, Yoo E, Jung H, Jang H (2016). Removal ratio of gaseous toluene and xylene transported from air to root zone via the stem by indoor plants. Environ Sci Pollut Res.

[CR34] Kim KJ, Jeong MI, Lee DW, Song JS, Kim HD, Yoo EH, Jeong SJ, Han SW, Kays SJ, Lim Y-W (2010). Variation in formaldehyde removal efficiency among indoor plant species. HortScience.

[CR35] Kim KJ, Jung HH, Seo HW, Lee JA, Kays SJ (2014). Volatile toluene and xylene removal efficiency of foliage plants as affected by top to root zone size. HortScience.

[CR36] Kim T-S, Hong S-Y, Kim J-E, Lim H-H, Shin H-S (2012). Simultaneous determination of 37 volatile organic compounds at ng/L concentration level in surface water by HS-SPME-GC/MS. Anal Sci Technol.

[CR37] Lawson SJ, Galbally IE, Powell JC, Keywood MD, Molloy SB, Cheng M, Selleck PW (2011). The effect of proximity to major roads on indoor air quality in typical Australian dwellings. Atmos Environ.

[CR38] Lewis AC (2018). The changing face of urban air pollution. Science.

[CR39] Liu C, Zhang N, Sun L, Gao W, Zang Q, Wang X (2022). Potted plants and ventilation effectively remove pollutants from tobacco smoke. Int J Low-Carbon Technol.

[CR40] Mann HS, Crump D, Brown V (2001). Personal exposure to benzene and the influence of attached and integral garages. J R Soc Promot Heal.

[CR41] Martinez Arbizu P (2017). pairwiseAdonis: pairwise multilevel comparison using adonis. R package version.

[CR42] Matheson S, Fleck R, Irga P, Torpy F (2023). Phytoremediation for the indoor environment: a state-of-the-art review. Rev Environ Sci Biotechnol.

[CR43] Micyus NJ, McCurry JD, Seeley JV (2005). Analysis of aromatic compounds in gasoline with flow-switching comprehensive two-dimensional gas chromatography. J Chromatogr A.

[CR44] Mikkonen A, Li T, Vesala M, Saarenheimo J, Ahonen V, Kärenlampi S, Tervahauta A (2018). Biofiltration of airborne VOC s with green wall systems—Microbial and chemical dynamics. Indoor Air.

[CR45] Mitchell CS, Zhang J, Sigsgaard T, Jantunen M, Lioy PJ, Samson R, Karol MH (2007). Current state of the science: health effects and indoor environmental quality. Environ Health Perspect.

[CR46] Morgan AL, Torpy FR, Irga PJ, Fleck R, Gill RL, Pettit T (2022). The botanical biofiltration of volatile organic compounds and particulate matter derived from cigarette smoke. Chemosphere.

[CR47] Mosaddegh MH, Jafarian A, Ghasemi A, Mosaddegh A (2014). Phytoremediation of benzene, toluene, ethylbenzene and xylene contaminated air by D. deremensis and O. microdasys plants. J Environ Health Sci Eng.

[CR48] Müller, H. W. a. R. F. a. L. H. a. K. (2022). dplyr: a grammar of data manipulation. In https://cran.r-project.org/package=dplyr

[CR49] Neghab M, Hosseinzadeh K, Hassanzadeh J (2015). Early liver and kidney dysfunction associated with occupational exposure to sub-threshold limit value levels of benzene, toluene, and xylenes in unleaded petrol. Saf Health Work.

[CR50] Nelson RD, Lide DR, Maryott AA (1967). Selected values of electric dipole moments for molecules in the gas phase.

[CR51] Odewabi A, Ogundahunsi O, Oyalowo M (2014). Effect of exposure to petroleum fumes on plasma antioxidant defense system in petrol attendants. Br J Pharmacol Toxicol.

[CR52] Organization, W. H (2010). WHO guidelines for indoor air quality: selected pollutants.

[CR53] Orwell RL, Wood RA, Burchett MD, Tarran J, Torpy F (2006). The potted-plant microcosm substantially reduces indoor air VOC pollution: II. Laboratory study. Water Air Soil Pollut.

[CR54] Pettit T, Irga P, Torpy F (2018). Functional green wall development for increasing air pollutant phytoremediation: substrate development with coconut coir and activated carbon. J Hazard Mater.

[CR55] Pettit T, Irga P, Torpy F (2019). The in situ pilot-scale phytoremediation of airborne VOCs and particulate matter with an active green wall. Air Qual Atmos Health.

[CR56] Pettit T, Bettes M, Chapman A, Hoch L, James N, Irga P, Torpy F, Plants, & Group, E. Q. R (2019). The botanical biofiltration of VOCs with active airflow: is removal efficiency related to chemical properties?. Atmos Environ.

[CR57] Prodanovic V, Hatt B, McCarthy D, Zhang K, Deletic A (2017). Green walls for greywater reuse: understanding the role of media on pollutant removal. Ecol Eng.

[CR58] Prodanovic V, Zhang K, Hatt B, McCarthy D, Deletic A (2018). Optimisation of lightweight green wall media for greywater treatment and reuse. Build Environ.

[CR59] Setsungnern A, Treesubsuntorn C, Thiravetyan P (2017). The influence of different light quality and benzene on gene expression and benzene degradation of Chlorophytum comosum. Plant Physiol Biochem.

[CR60] Sriprapat W, Suksabye P, Areephak S, Klantup P, Waraha A, Sawattan A, Thiravetyan P (2014). Uptake of toluene and ethylbenzene by plants: removal of volatile indoor air contaminants. Ecotoxicol Environ Saf.

[CR61] Sriprapat W, Thiravetyan P (2013). Phytoremediation of BTEX from indoor air by Zamioculcas zamiifolia. Water Air Soil Pollut.

[CR62] Sriprapat W, Thiravetyan P (2016). Efficacy of ornamental plants for benzene removal from contaminated air and water: effect of plant associated bacteria. Int Biodeterior Biodegradation.

[CR63] Suárez-Cáceres GP, Fernández-Cañero R, Fernández-Espinosa AJ, Rossini-Oliva S, Franco-Salas A, Pérez-Urrestarazu L (2020). Volatile organic compounds removal by means of a felt-based living wall to improve indoor air quality.

[CR64] Teiri H, Pourzamani H, Hajizadeh Y (2018). Phytoremediation of VOCs from indoor air by ornamental potted plants: a pilot study using a palm species under the controlled environment. Chemosphere.

[CR65] Terrés IMM, Miñarro MD, Ferradas EG, Caracena AB, Rico JB (2010). Assessing the impact of petrol stations on their immediate surroundings. J Environ Manag.

[CR66] Thomas KW, Pellizzari E, Clayton C, Perritt R, Dietz R, Goodrich R, Nelson W, Wallace L (1993). Temporal variability of benzene exposures for residents in several New Jersey homes with attached garages or tobacco smoke. J Expo Anal Environ Epidemiol.

[CR67] Torpy F, Clements N, Pollinger M, Dengel A, Mulvihill I, He C, Irga P (2018). Testing the single-pass VOC removal efficiency of an active green wall using methyl ethyl ketone (MEK). Air Qual Atmos Health.

[CR68] Torpy F, Irga P, Moldovan D, Tarran J, Burchett M (2013). Characterization and biostimulation of benzene biodegradation in the potting-mix of indoor plants. J Appl Hortic.

[CR69] Torpy F, Zavattaro M, Irga P (2017). Green wall technology for the phytoremediation of indoor air: a system for the reduction of high CO_2_ concentrations. Air Qual Atmos Health.

[CR70] Torpy FR, Pettit T, Irga PJ (2018). Applied horticultural biotechnology for the mitigation of indoor air pollution. J People, Plants Environ.

[CR71] Treesubsuntorn C, Suksabye P, Weangjun S, Pawana F, Thiravetyan P (2013). Benzene adsorption by plant leaf materials: effect of quantity and composition of wax. Water Air Soil Pollut.

[CR72] Treesubsuntorn C, Thiravetyan P (2012). Removal of benzene from indoor air by Dracaena sanderiana: effect of wax and stomata. Atmos Environ.

[CR73] Tsai P-Y, Weisel CP (2000). Penetration of evaporative emissions into a home from an M85-fueled vehicle parked in an attached garage. J Air Waste Manage Assoc.

[CR74] Wang S, Yuan B, Wu C, Wang C, Li T, He X, ... & Shao M (2022) Oxygenated VOCs as significant but varied contributors to VOC emissions from vehicles. Atmospheric Chemistry & Physics Discussions

[CR75] Warden H, Richardson H, Richardson L, Siemiatycki J, Ho V (2018). Associations between occupational exposure to benzene, toluene and xylene and risk of lung cancer in Montréal. Occup Environ Med.

[CR76] Wickham MH (2014) Package “ggplot2” Type Package Title An implementation of the Grammar of Graphics

[CR77] Wickham H (2020). tidyr: Tidy Messy Data. R package version 1.1. 2 (1.1. 2). CRAN. R-project.org/package=tidyr

[CR78] Wolverton B, McDonald R (1982). Foliage plants for removing formaldehyde from contaminated air inside energy-efficient homes and future space stations.

[CR79] Wolverton BC, McDonald RC, Watkins E (1984). Foliage plants for removing indoor air pollutants from energy-efficient homes. Econ Bot.

[CR80] Wood RA, Burchett MD, Alquezar R, Orwell RL, Tarran J, Torpy F (2006). The potted-plant microcosm substantially reduces indoor air VOC pollution: I. Office field-study. Water Air Soil Pollut.

[CR81] Yue T, Yue X, Chai F, Hu J, Lai Y, He L, Zhu R (2017). Characteristics of volatile organic compounds (VOCs) from the evaporative emissions of modern passenger cars. Atmos Environ.

