# Supplementary figures and images for: Fuelling phytoremediation: gasoline degradation by green wall systems—a case study
Source: Environ Sci Pollut Res Int. 2023 Nov 2;30(56):118545–55. doi: 10.1007/s11356-023-30634-1 (PMC10698092; doi:10.1007/s11356-023-30634-1)

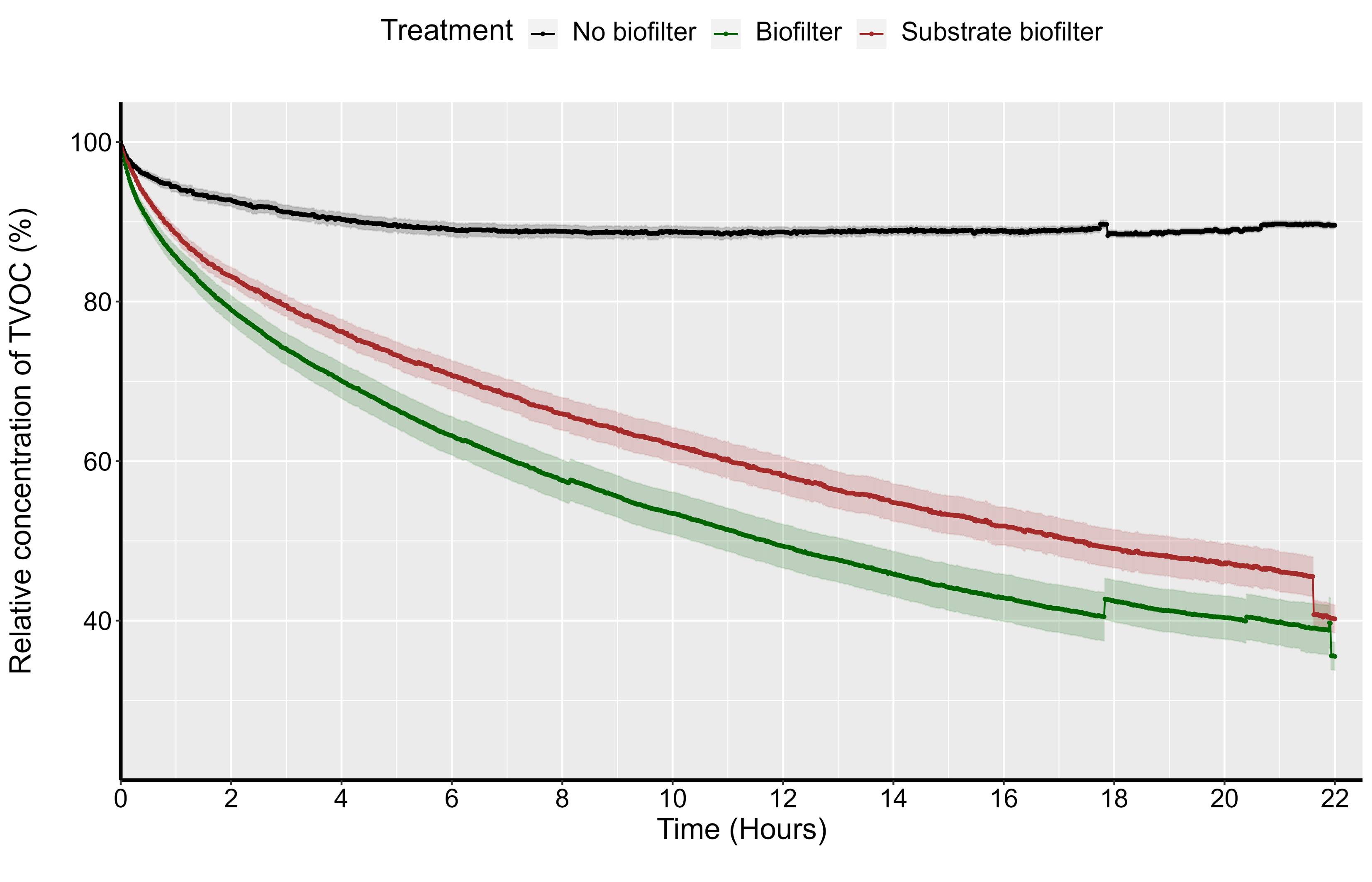

Supplement: Supplementary file 1 — Figure S1. Relative TVOC drawdown for each treatment over 22 h. Error bands represent the SEM (PNG 653 kb) [file 11356_2023_30634_Fig5_ESM.png]

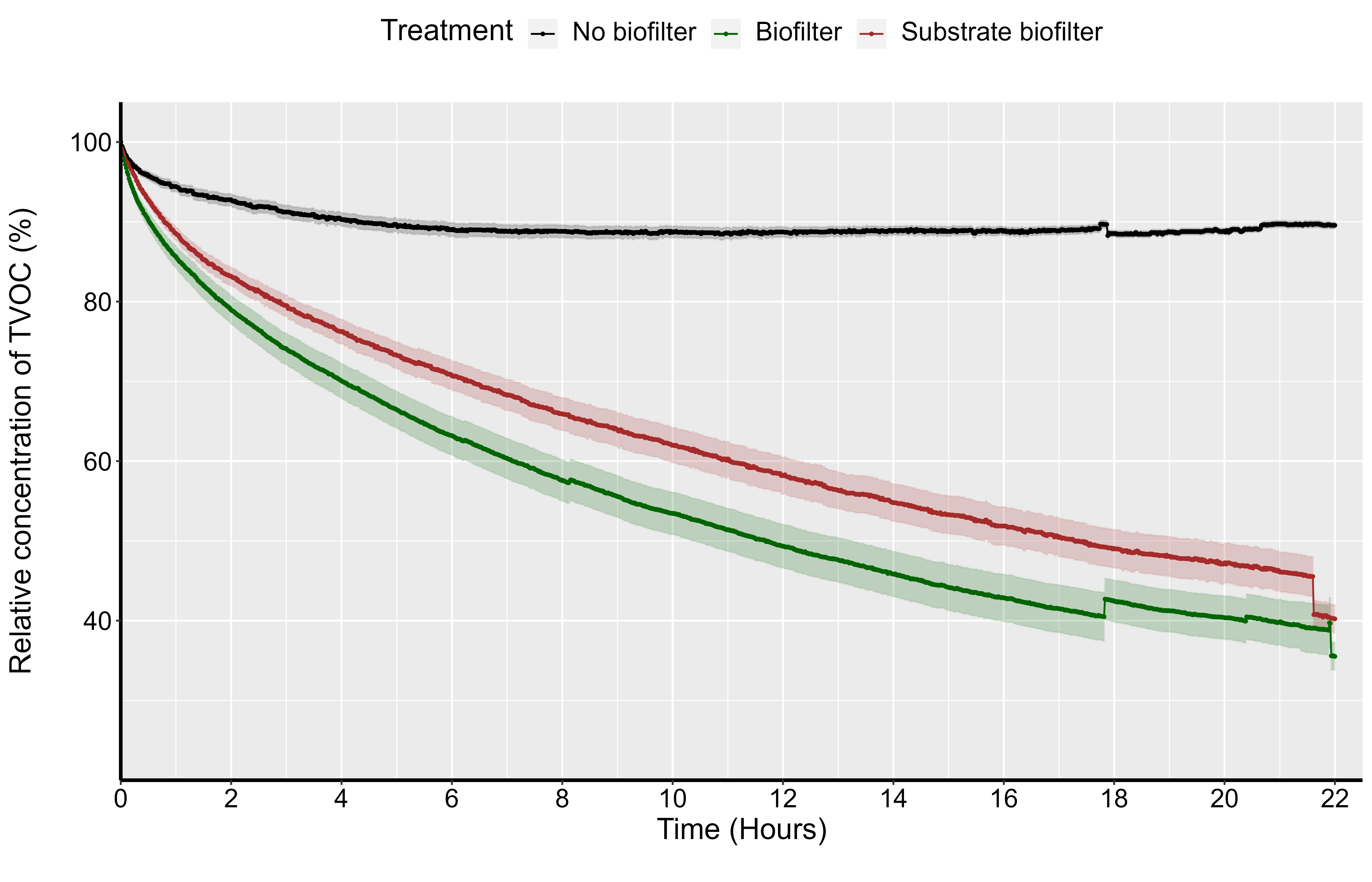

Supplement: Supplementary file 2 — High resolution image (TIF 2.25 mb) [file 11356_2023_30634_MOESM1_ESM.tif]
